# Letter to the Editor: ‘Exploring barriers to expanding medical training numbers in England: A national survey of medical education directors’

**DOI:** 10.1016/j.clinme.2026.100561

**Published:** 2026-02-18

**Authors:** Emma Paoletti

**Affiliations:** Care of the Elderly Rotation, Wrexham Maelor Hospital, Wrexham, Wales

Dear Editor,

I have read the article by Batavanis *et al* titled ‘*Exploring barriers to expanding medical training numbers in England: A national survey of medical education directors’*^1^ with great interest as it is a significant and very relevant issue in medical and surgical training in the UK.

This study highlights the need for an expanding medical workforce while simultaneously emphasising the need for increased funding and educational resources for trainees in England.

As stated in the article, measures are being taken to increase the medical workforce in the coming years, including raising medical school places, permitting professionals to qualify through alternative programmes such as apprenticeships and increasing junior doctor posts nationwide.

However, I believe the article lacks to mention an increasing problem in medical and surgical training progression for already qualified doctors, which would only be exacerbated by an increasing number of medical graduates and junior doctors.

In fact, current junior doctors are facing considerable competition when applying to specialties and trainee training, with application ratios ranging from 0.53 to 112.13,^2^ as per 2024 data provided by NHS England.^2^ This highlights the need for an increase in the number of available posts at middle and higher levels of training, more so than medical graduates and junior doctors. In fact, in 2025 all eligible foundation doctor applicants were allocated a job.^3^ The issue lies with the fact that many medical graduates are currently or unable to progress in their careers and training due to a low number of specialty training posts.

Therefore, I believe that while increasing the numbers of medical students, junior doctors and primary care posts would be important in increasing the medical workforce over time, this would need to be accompanied by an increase in the number of higher specialty training posts to allow medical professionals to progress through their careers while increasing the number of medical school places and junior doctor posts.

## Funding

This research did not receive any specific grant from funding agencies in the public, commercial, or not-for-profit sectors.

## CRediT authorship contribution statement

**Emma Paoletti:** Writing – review & editing, Writing – original draft, Validation, Software, Resources, Conceptualization.

## Declaration of competing interest

None.
